# Effects of avian *Plasmodium* exposure on the microbiota of *Culex pipiens*

**DOI:** 10.1038/s41598-025-22774-w

**Published:** 2025-11-06

**Authors:** Marta Garrigós, Olaya García-Ruiz, Charlotte R. Enkvist, María José García-López, Isabel Moreno-Indias, María José Ruiz-López, Jesús Veiga, Jordi Figuerola, Elin Videvall, Josué Martínez-de la Puente

**Affiliations:** 1 Department of Conservation Biology and Global Change, Doñana Biological Station (EBD), CSIC, Sevilla, Spain; 2 CIBER de Epidemiología y Salud Pública (CIBERESP), Madrid, Spain; 3 Department of Ecology and Genetics, Uppsala University, Uppsala, Sweden; 4 Department of Endocrinology and Nutrition, Instituto de Investigación Biomédica de Málaga (IBIMA), Málaga, Spain; 5 Hospital Universitario Virgen de la Victoria, Málaga, Spain; 6 Facultad de Medicina, Universidad de Málaga, Málaga, Spain; 7 CIBER de Fisiopatología de la Obesidad y Nutrición (CIBEROBN), Madrid, Spain

**Keywords:** Avian malaria, Bacteria, Microbiome, Mosquitoes, Vector-borne pathogens, Microbial ecology, Entomology

## Abstract

Malaria parasites (*Plasmodium* spp.) are mosquito-borne parasites that infect humans and wildlife. Several studies support the role of mosquito microbiota as a major driver of *Plasmodium* transmission, although studies on wildlife malaria are typically neglected. Here, we used a 16S rRNA metabarcoding approach to assess whether the exposure to avian *Plasmodium* parasites affects the microbiota of their natural vector, *Culex pipiens*. Mosquitoes, captured in the field as larvae and grown in the laboratory, were allowed to feed on house sparrows (*Passer domesticus*) naturally infected with *Plasmodium relictum* (lineage SGS1) and uninfected birds. We analyzed the microbiota composition of the abdomens of individual mosquitoes and found 2,006 Amplicon Sequence Variants (ASVs). *Culex pipiens’* microbiota was dominated by bacteria of the genus *Wolbachia*, followed by the genera *Stenotrophomonas* and *Faecalibacterium*. We observed no difference in alpha nor beta diversity between mosquitoes that fed on *Plasmodium-*infected birds (exposed mosquitoes) and those that fed on uninfected birds (unexposed mosquitoes). However, exposed mosquitoes had a higher relative abundance of bacteria of the family *Bacteroidaceae* and the genus *Bacteroides* than the unexposed mosquitoes. Excluding the intracellular endosymbiont *Wolbachia* from the analyses, we obtained similar results, and also found a higher relative abundance of bacteria of the family *Rikenellaceae* in exposed mosquitoes. A pathway enrichment analysis based on KEGG annotations revealed that the bacterial community in exposed mosquitoes was enriched in pathways mainly related to biosynthesis and metabolism. Our results suggest that *Cx. pipiens* exposed to avian *Plasmodium* have slightly different microbiota composition, although further research is needed to establish the causality of these effects.

**Supplementary Information:**

The online version contains supplementary material available at 10.1038/s41598-025-22774-w.

## Introduction

Malaria, caused by parasites of the genus *Plasmodium*, produces around half a million human fatalities annually^1^. In addition to humans, other non-human vertebrates are infected by *Plasmodium* parasites, causing diseases with ecological and evolutionary consequences^2^. Most *Plasmodium* parasites require mosquitoes to complete their life cycle and be transmitted from an infected individual to a new host. The development of *Plasmodium* in mosquitoes is modulated by abiotic and biotic factors, including intrinsic factors such as the composition of the digestive tract bacterial microbiota of mosquitoes (hereafter gut microbiota)^3,4^. Mosquitoes acquire most of their gut microbiota during the aquatic larval stage from their breeding sites^4^, while a small proportion of this microbiota is transferred to the adult stage through transstadial transmission^5^. The gut microbiota of adults is modified by their feeding sources, including plant sugars, water and, in the case of females, the blood meals from vertebrate hosts^6,7^.

The mosquito microbiota interacts with *Plasmodium* through direct mechanisms, producing metabolites with antiparasitic properties and reactive oxygen species^8^, but also through indirect mechanisms. The microbiota not only enhances the activation of mosquito immune responses, it also favors the formation of physical barriers against the parasite (e.g., the gut peritrophic matrix)^9^. Disruptions of the microbiota affect the susceptibility of mosquitoes to *Plasmodium*, as supported by studies with different malaria models^10,11^. However, contradictory results have been found depending on the bacteria-parasite-mosquito system studied. For example, bacteria of the genera *Comamonas*, *Acinetobacter*, *Pseudomonas*, *Pantoea*, *Serratia*, *Elizabethkingia*,* Enterobacter* and *Chromobacterium* totally or partially blocked the development of *Plasmodium falciparum* in *Anopheles gambiae*^8,12,13^. *Wolbachia* increases the susceptibility of *Culex pipiens* mosquitoes to *Plasmodium relictum*, by increasing the prevalence of the parasite’s infective stage^14^. On the other hand, *Plasmodium* infection can also affect the microbiota composition of mosquitoes. Aželytė et al.^15^ found significant differences in the relative abundance of 20 bacterial genera between *P. relictum*-infected and uninfected *Culex quinquefasciatus* mosquitoes, using mosquito colonies and experimentally infected canaries as parasite donors. However, further studies are necessary to reveal the interactions between mosquito microbiota and avian *Plasmodium*, especially using natural parasite-mosquito assemblages, given the strong impact that the environment may have on the microbiota composition of mosquitoes^16,17^.

*Plasmodium relictum* is a generalist malarial parasite that infects bird species worldwide and is considered one of the most dangerous invasive species due to its negative effects on bird populations in invaded areas^2,18^. *Culex pipiens* is a well-known vector of avian *Plasmodium*, including *P. relictum*^19^. Here, we performed a proof-of-concept study to identify the potential associations between mosquito microbiota and exposure to avian *Plasmodium* using a natural host-parasite-vector assemblage. Using a 16S rRNA metabarcoding approach, we compared the abdominal microbiota (hereafter referred to as microbiota in the context of this study) of *Cx. pipiens* females 21 days after feeding on *P. relictum-*infected house sparrows with that of females fed on uninfected sparrows.

## Results

We initially selected 64 mosquitoes to be analyzed for microbiota composition. There was no amplification of the 16S rRNA gene for five samples and fewer than 1,000 reads were obtained for 39 samples. Therefore, after the data filtering steps, 20 samples were included in the final analysis, corresponding to 13 unexposed mosquitoes (fed on uninfected birds) and seven exposed mosquitoes (fed on birds infected by *P. relictum -* lineage SGS1). Two of the seven exposed mosquitoes tested positive for parasite infection in the head and thorax, and five were negative.

**Fig. 1:**
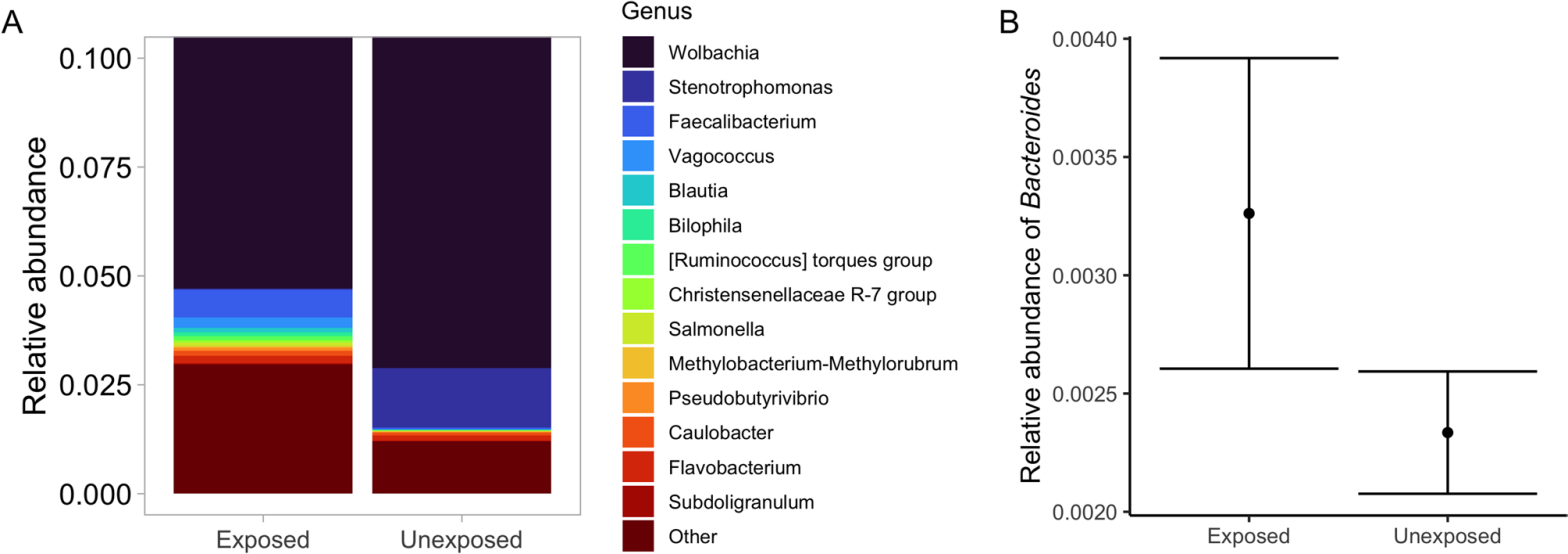
Abdominal microbiota of mosquitoes fed on *Plasmodium relictum-*infected (exposed) and uninfected birds (unexposed). (**A**) Relative abundance of the 15 most abundant genera of bacteria in exposed and unexposed mosquitoes. The y-axis shows a zoomed-in version of the relative abundance of bacterial genera from 0 to 0.1. From 0.1 to 1, the relative abundance corresponds solely to the *Wolbachia* genus (not pictured). (**B**) Relative abundance of the genus *Bacteroides* in the abdominal microbiota of exposed and unexposed mosquitoes. Bars correspond to standard errors.

The analyses of the microbiota of these 20 *Cx. pipiens* resulted in a total of 6,911,775 reads, ranging from 1,134 to 1,816,458 (average = 345,588.75). Overall, 2,006 Amplicon Sequence Variants (ASVs) were identified, classified to 23 different phyla and, at least, 46 classes, 115 orders, 199 families, and 405 genera (Fig S1-S5). In terms of relative abundance, the microbiota of *Cx. pipiens* was dominated by the phylum *Proteobacteria* (average: 94.86%, SD = 0.124), followed by *Bacillota* (formerly *Firmicutes*) (average: 3.51%, SD = 0.110), *Actinobacteriota* (average: 0.79%, SD = 0.010), *Cyanobacteria* (average: 0.32%, SD = 0.006), and *Bacteroidota* (average: 0.31%, SD = 0.004) (Fig S1). Most reads (96.56%) corresponded to the genus *Wolbachia* (family *Anaplasmataceae*), which was the most abundant taxon in all samples (average relative abundance = 91.30%, SD = 0.141), followed by *Stenotrophomonas* (family *Xanthomonadaceae*), *Faecalibacterium* (family *Ruminococcaceae*), *Vagococcus* (family *Enterococcaceae*), *Blautia* (family *Lachnospiraceae*), *Bilophila* (family *Desulfovibrionaceae*), and *Ruminococcus* (family *Ruminococcaceae*), all of them with an average relative abundance below 2% (Fig. 1A) (see Supplementary Table S1 for the relative abundance of all genera).

**Fig. 2:**
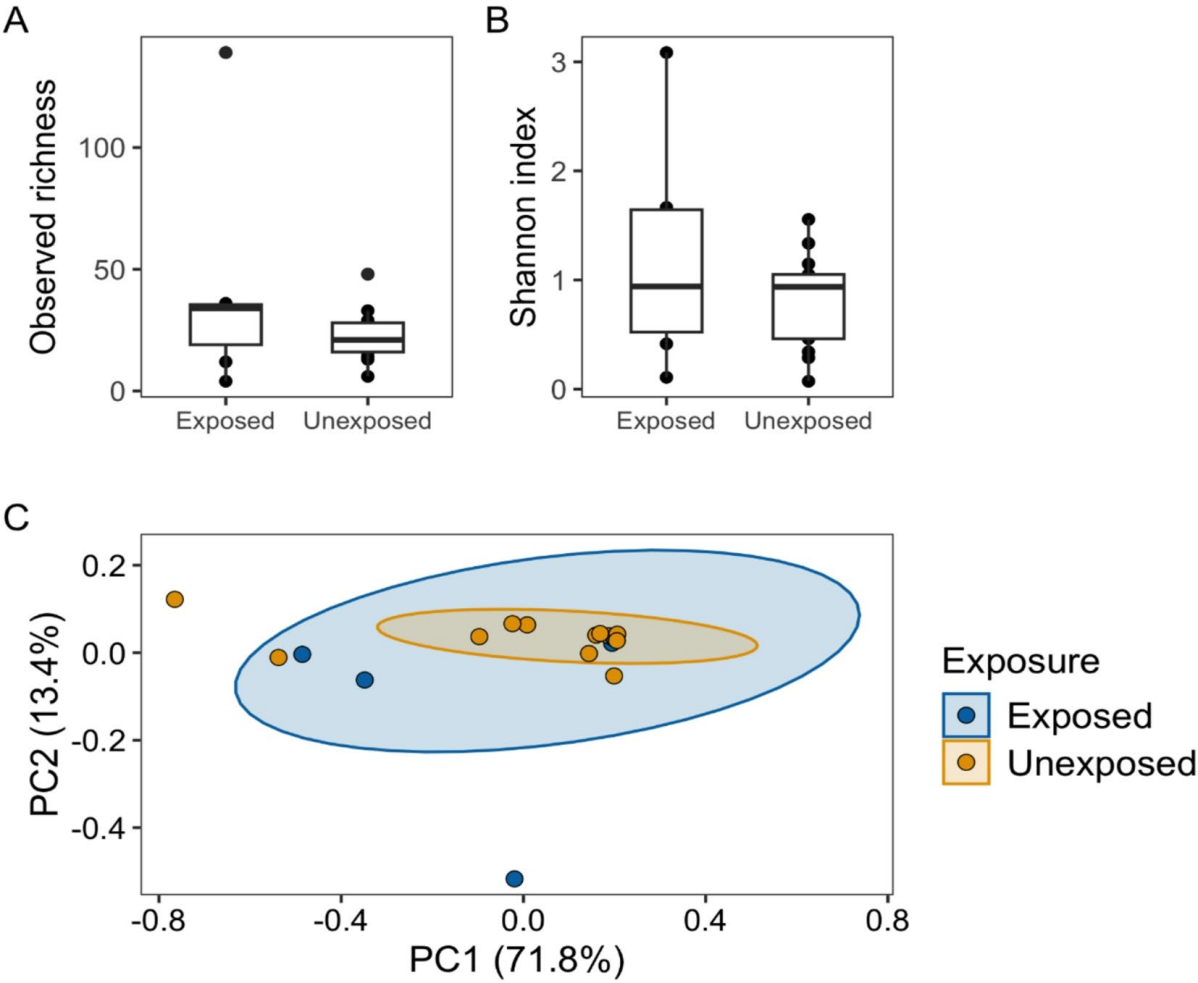
Diversity of the abdominal microbiota of mosquitoes fed on *Plasmodium relictum-*infected (exposed) and uninfected birds (unexposed). (**A**) Distribution of observed richness in the microbiota of exposed and unexposed mosquitoes. The horizontal lines represent the median observed richness of each group. The boxes delimit the upper (75%) and lower (25%) quartiles, and the vertical lines go from the lower and upper quartiles to the minimum or maximum values. (**B**) Distribution of the Shannon index estimate in the microbiota of exposed and unexposed mosquitoes. (**C**) Principal Co-ordinates Analysis (PCoA) for Bray-Curtis distance matrix (relative abundance) in the microbiota of exposed and unexposed mosquitoes. PC1 and PC2 axes refer to the percentage of variation explained by each of the two selected main coordinate axes.

We did not find significant differences in the alpha diversity of the microbiota between exposed and unexposed mosquitoes, including richness (Kruskal–Wallis (K–W): χ² = 1.146, Degrees of freedom (Df) = 1, *P* = 0.284) and Shannon index (K–W: χ² = 0.566, Df = 1; *P* = 0.451) (Fig. 2A and B). Similarly, we did not find significant differences in the beta diversity of the microbiota of exposed and unexposed mosquitoes (PERMANOVA: F = 0.381, Df = 1, *P* = 0.77) (Fig. 2C). The microbiota of exposed and unexposed mosquitoes shared a total of 126 families (63.32%) and 204 (50.37%) genera, with the remaining bacteria being exclusively found in one of the two experimental groups (Fig S6; Supplementary Table S2). Moreover, a differential abundance analysis showed that the microbiota of exposed mosquitoes had a higher relative abundance of the family *Bacteroidaceae* (ancombc2: Log Fold Change (LFC) = 3.14; *P* = 0.014) and the genus *Bacteroides* (ancombc2: LFC = 3.14; *P* = 0.024) than that of unexposed mosquitoes (Fig. 1B).

We repeated the analyses after excluding ASVs belonging to the genus *Wolbachia*. After filtering for number of reads, this new dataset included 18 mosquitoes (13 unexposed and five exposed). The results were similar to those obtained when *Wolbachia* was included in the analyses. Again, no differences were found in the alpha diversity, including richness (K–W: χ² = 2.1885, Df = 1, *P* = 0.139) and Shannon index (K–W: χ² = 1.7709, Df = 1, *P* = 0.183) (Fig S7A and B), and beta diversity (PERMANOVA: F = 0.658, Df = 1, *P* = 0.87), between the microbiota of exposed and unexposed mosquitoes (Fig S7C). However, the microbiota of exposed mosquitoes had a higher relative abundance of the bacterial family *Bacteroidaceae* (ancombc2: LFC = 2.82; *P* = 0.019) and the genus *Bacteroides* (ancombc2: LFC = 2.82; *P* = 0.034) than that of unexposed mosquitoes. In addition, the microbiota of exposed mosquitoes also had a higher relative abundance of the family *Rikenellaceae* (ancombc2: LFC = 1.93; *P* = 0.048).

A total of 7,178 Kyoto Encyclopedia of Genes and Genomes (KEGG) orthologs were identified from the bacterial community of *Cx. pipiens*. The KEGG pathway analysis revealed that the microbiota of exposed mosquitoes differed in 70 pathways (|LFC| > 2, 0.98%) compared with the microbiota of unexposed mosquitoes. These pathways were related to ribosome, two-component system, ABC transporters, calcium signaling pathway, focal adhesion, oxidative phosphorylation, peptidoglycan biosynthesis, and tryptophan metabolism, among others (Supplementary Table S3). After excluding *Wolbachia*, the microbiota of exposed mosquitoes differed in 122 pathways (1.70%). These 122 differentially abundant pathways included all pathways previously found when *Wolbachia* was considered in the analysis, together with pathways related to metabolism of different biomolecules like galactose, starch and sucrose, D-amino acid, lysine, cysteine and methionine, and pyruvate. Additional differentially abundant pathways included the phosphotransferase system (PTS) and DNA replication, among others (Supplementary Table S3).

## Discussion

The characterization of the abdominal microbiota of adult *Cx. pipiens* mosquito females emerged in the laboratory from field-collected larvae showed the dominance of the phylum *Proteobacteria*. Other bacteria of the phyla *Bacillota*, *Bacteroidetes*, *Actinobacteria*, and *Cyanobacteria* were also commonly found, as previously reported in studies using wild mosquito populations^6,20^. *Wolbachia* was the most abundant genus in the samples representing, on average, more than 90% of the mosquito abdominal microbiota. This bacterium typically dominates the general microbial community of many wild mosquitoes, including *Cx. pipiens*^21–23^. *Wolbachia* spp. are endosymbiotic bacteria present in different mosquito tissues, with particularly high abundance in reproductive organs^24^, but also found in other tissues like the digestive tract^25^. The next most abundant genera in our samples were *Stenotrophomonas*, *Faecalibacterium*, *Vagococcus*, *Blautia*, *Bilophila*, and *Ruminococcus*. A similar bacterial community was previously found in whole-body *Aedes albopictus* samples from Madagascar, which was dominated by *Wolbachia*, but also presented bacteria of the genera *Blautia*, *Faecalibacterium*, and *Ruminococcus*^21^. These bacteria, together with *Stenotrophomonas*, are part of the microbiota of different mosquito species in other geographical areas^26–28^. The microbiota found in the related species *Cx. perexiguus* from southern Spain is, however, very different, because *Wolbachia* is virtually absent^29^. The differences in the microbiota composition between species from the same region support the relevance of mosquito species identity as a major driver of the composition of mosquito microbiota in the wild^22^. Because the microbiota of mosquitoes may vary between body parts, future studies focusing on the characterization of the gut microbiota may dissect the posterior midgut of mosquitoes, and extract DNA from this tissue alone, instead of using the whole abdomen, as we did.

We found a higher relative abundance of bacteria of the family *Bacteroidaceae* and the genus *Bacteroides* in the abdominal microbiota of exposed mosquitoes compared to unexposed ones. The genus *Bacteroide*s has been associated with a higher susceptibility to *Plasmodium* in humans and rodents^30^. In mosquitoes, the bacterium *Bacteroides vulgatus* was enriched in wild *Aedes* mosquitoes infected by Zika virus^28^. Our results were highly consistent when both including and excluding *Wolbachia* from our dataset. In addition, we identified a higher relative abundance of the *Rikenellaceae* bacteria in exposed mosquitoes, when *Wolbachia* was excluded from the analyses. As a member of insect gut microbiota, the *Rikenellaceae* family plays a role in vitamin synthesis^31^, and is highly abundant in mosquitoes from, for instance, Brazil, especially during the larval stage^32^. However, to our knowledge, it has not been previously associated with *Plasmodium* parasites in mosquitoes. In addition to differences in mosquito microbiota, we also identified differentially abundant KEGG orthologs (KOs) between the microbiota of exposed and unexposed mosquitoes, obtaining similar results with and without *Wolbachia*. The microbiota of exposed mosquitoes showed an enrichment in pathways related to metabolism. Furthermore, despite minor whole-community differences in the microbiota of mosquitoes according to the experimental procedure, the microbiota of exposed mosquitoes was generally enriched with the two-component system, a signal transduction pathway in bacteria regulating responses to environmental stimuli^33^. These metabolic associations should be further explored by assessing the correlation between the mosquito microbiota and transcriptomic responses.

Due to the cross-sectional experimental approach, we were not able to determine if differences in mosquito microbiota were caused by or a consequence of avian *Plasmodium* exposure. However, the fact that mosquitoes exposed to infected and uninfected birds had the same origin, potentially harboring a similar microbiota, suggests that differences could potentially be caused by parasite exposure. *Culex pipiens* can develop infective parasite stages after feeding on birds with low *Plasmodium* parasitemia levels (diagnosed negative according to blood smear observation)^34^, although some exposed mosquitoes may remain uninfected^35^. Indeed, we only detected parasite DNA in two of the seven exposed mosquitoes. In addition, the pattern found here could partly reflect differences in the characteristics of avian blood meals from infected and uninfected birds. Red cell density, metabolites, and immune factors in the blood are likely to differ between infected and uninfected birds^36,37^, which can affect mosquito physiology^38^, potentially leading to shifts in the composition and function of the microbiota. These changes could affect mosquito resistance to pathogens, lifespan and fecundity^10^. Another source of variation in the mosquito microbiota could potentially be due to differences in the bacterial communities of the skin and feathers of birds used as donors, which might be ingested by mosquitoes during blood-feeding.

Finally, in spite of using organisms from the field including wild house sparrows naturally infected by the local avian *Plasmodium* strain and field-collected vectors as larvae, our results could be affected by the conditions used during the experiments. These include, for example, the exposure to bacteria found in the laboratory during mosquito development or the food sources used for their maintenance. This experimental setup is unlikely to exactly reflect the conditions found in the wild. In addition, including mosquitoes that received different concentrations of sugar in their diet could affect the results, although this variable was confirmed to not be significantly correlated with bacterial alpha and beta diversity in this study (Supplementary Material 1) and was corrected for in the differential abundance models.

## Conclusion

Our results support a minor but significant difference in the *Cx. pipiens* mosquito abdominal microbiota after exposure to avian *Plasmodium-*infected birds, which could be detected in the metabolic pathways of bacteria. In order to confirm causality of this relationship, experimental manipulation of the mosquito microbiota, with a special focus on the family *Bacteroidaceae*, prior to the exposure to parasite infections, will be necessary.

## Materials and methods

### Experimental setup

Wild juvenile house sparrows (*Passer domesticus*) (*n* = 20) were captured with mist nets in August 31st and September 9th 2021, in Trigueros (Huelva province, Spain; 37°19’33.7"N, 6°47’45.6"W). Each house sparrow was ringed, weighed and the wing length measured before being brought into captivity to the animal facilities of the Doñana Biological Station. All procedures were conducted in accordance with relevant guidelines and regulations, including compliance with national legal requirements for animal experimentation (Article 34 RD 53/2013). Following the conclusion of the experiment, the birds were released at the original capture site. The protocol was approved by the Animal Experimentation Committee (ref. 12/07/219/129). Reporting of methods and results adheres to the ARRIVE guidelines. A blood sample was collected from the jugular vein of each bird using a sterile syringe to molecularly identify infection status and the parasite lineage identity. A drop of blood was used to make a blood smear. Genomic DNA was extracted from blood samples using Maxwell^®^ 16 LEV Blood DNA Kit (Promega, Madison, WI, USA)^39^ and the *Plasmodium* infection status and parasite identity were assessed following the protocol by Hellgren et al.^40^. This method has a sensitivity of, at least, one parasite per 100,000 host blood cells. The amplified products of the positive samples were sequenced on both strands using Capillary Electrophoresis Sequencing by Macrogen (Madrid, Spain). The sequences were analyzed using Geneious v. 2020.0.3^41^ and assigned to lineages using the blast tool in MalAvi^42^. The blood smears were used to visually check the infection status and to quantify the levels of parasitemia in each bird using a light microscope ZEISS Axioscope 5. We observed no parasite cells in 10,000 erythrocytes in uninfected birds and one mature parasite cell per 10,000 erythrocytes in infected birds. After molecular and microscopic analysis, six birds were chosen for further procedures: three birds infected by *P. relictum* lineage SGS1 and three uninfected birds. These birds were verified to be uninfected by other blood parasites of the related genera *Haemoproteus* and *Leucocytozoon*. At the end of the experiments, birds were blood sampled again to test the presence of infection and quantify the parasitemia levels. PCR tests confirmed that the three uninfected birds remained negative for avian malaria, and the three infected birds continued to test positive for *Plasmodium*. However, blood smears from the infected birds no longer presented parasites, suggesting that the intensity of parasitemia might have decreased to fewer than one parasite cell per 10,000 erythrocytes.

Mosquito larvae were collected in September and October of 2021 from a single pond in La Rinconada (Seville, province Spain; 37º 29′ 16.8′′ N, 5º 58′′ 44.4′′ W). Once in the laboratory, larvae were placed in plastic trays with dechlorinated water and fed *ad libitum* with Hobby-Mikrozell 20 ml/22 g (Dohse Aquaristik, Gelsdorf, Germany) and Hobby-Liquizell 50 ml (Dohse Aquaristik). Larvae and adult mosquitoes were maintained under controlled conditions (26 °C ± 1, 55–60% relative humidity (RH) and 12:12 light: dark photoperiod cycle). Recently emerged adult mosquitoes had access to 0.5 w/v% (0.5%) sucrose solution for a maximum of 24 h to increase their chances of survival^43^. Adult *Cx. pipiens* females were morphologically identified under a stereomicroscope following Schaffner et al.^44^. The females were then sorted into groups of equal numbers and placed into separate insect cages (BugDorm-43030 F, 32.5 × 32.5 × 32.5 cm) with three different sucrose concentrations: *ad libitum* access to 0.5%, 2% and 8% in the context of a related study (unpublished data). Cages were randomly placed at different positions in the climatic chambers to control for possible spatial bias. For each diet-group, seven-day-old female mosquitoes were allowed to feed overnight on either a *P. relictum-*infected bird or an uninfected bird. Each bird was placed in a cage containing approximately 100 mosquitoes. A minimum of eight days passed from bird capture (when parasite detection was recorded in peripheral blood samples) to mosquito exposure. Fully blood-engorged females were placed in separate cages according to the diet they received before and after the blood meal (0.5%, 2% or 8%) and the infection status of the bird they fed on (positive or negative). We used six different birds in this experiment, three *P. relictum-*infected and three uninfected birds. Each mosquito-diet group was exposed to a different pair of birds, consisting of one infected and one uninfected bird. We performed three replicates of this experiment; in each replicate, we rotated the bird pairs among the diet groups, so that all birds were exposed to mosquitoes from all sucrose diets. The mosquitoes included in the final analysis fed on five different house sparrows, including three *Plasmodium*-infected and two uninfected birds (Table 1). Mosquitoes were kept in the same climatic chamber for 21 days after feeding to allow parasite development until the sporozoite stage^45^. Mosquitoes were then frozen at -80 °C until molecular analyses.

**Table 1:** Total number of samples (N) included in the final analysis (after filtering by number of reads), according to replicate, bird ID, bird infection status, mosquito infection status and diet.

*N*	Replicate	Bird ID	Bird infection status	Mosquito infection status	Diet (% sugar)
3	A	178	Negative	–	0.5
3	A	182	Negative	–	2
2	C	178	Negative	–	8
4	E	178	Negative	–	2
1	A	165	Positive	Negative	2
1	C	164	Positive	Negative	8
1*	E	187	Positive	Negative	0.5
1	E	187	Positive	Positive	0.5
2*	E	164	Positive	Negative	2
1	E	164	Positive	Positive	2

*Groups for which one sample was removed in the analysis excluding *Wolbachia*.

### Molecular and bioinformatic analysis

Mosquitoes were sterilized individually by submerging them three times in 70% ethanol and once in sterilized water. Similar approaches have been previously used^21^, although this method may not fully eliminate external DNA^46^. Subsequently, mosquitoes were dissected to separate the head and thorax from the abdomen using two sterilized pipette tips in a sterilized petri dish. Genomic DNA from the head and thorax of mosquitoes was extracted using the Maxwell^®^ 16 LEV Blood DNA Kit (Promega, Madison, WI). We analyzed the *Plasmodium* infection status of mosquitoes following Hellgren et al.^40^.

The abdomens of 64 mosquitoes were used for individual microbiota characterization. The DNA was extracted using the DNeasy Blood & Tissue Kit ^®^ (QIAGEN, Hilden, Germany). The DNA from each mosquito was then used to build microbiota sequence libraries using the Ion 16S Metagenomics kit (Thermofisher, Waltham, Massachusetts, USA), which consists of primer pools that amplify multiple variable regions (V2, 3, 4, 6–7, 8 and 9) of the 16S ribosomal RNA gene. After generating amplicons, the Ion PlusTM Fragment Library Kit (Thermofisher, Waltham, Massachusetts, USA) was used to ligate individual barcoded adapters and synthesize separate libraries for each sample. Barcoded libraries from all the samples were pooled and templated on the automated Ion Chef system (Thermofisher, Waltham, Massachusetts, USA), followed by a 400 bp sequencing on the Ion S5 (Thermofisher, Waltham, Massachusetts, USA). The quality of the reads was assessed using FastQC (ver. 0.12.1)^47^ and MultiQC (ver. 1.17)^48^. The following analyses were performed in R (ver. 4.4.1)^49^ using Bioconductor packages. The *dada2* package (ver. 1.32.0)^50^ was used to filter and trim the raw reads, and to construct ASVs using the parameters for Ion Torrent data recommended by the developer (https://benjjneb.github.io/dada2/tutorial.html). The dada2 package was also used to assign each ASV to a taxonomic group based on the SILVA database (ver. 138.1)^51^. Reads were filtered, removing singletons, reads that lacked taxonomic assignment below phylum level, and non-bacterial, chloroplast, and mitochondrial ASVs using the *phyloseq* package (ver. 1.48.0)^52^. Samples with fewer than 1,000 reads were discarded. In addition, since bacteria of the genus *Wolbachia* are endosymbionts distributed across various mosquito tissues^24^, we repeated the analysis using the dataset excluding ASVs corresponding to this genus. In this additional analysis, we discarded samples with fewer than 500 reads, resulting in a final dataset of 18 individuals.

### Statistical analysis

We first performed an exploratory analysis of the microbiota composition according to the infection status of the mosquitoes (Supplementary Material 1; Figs S8A, S8B and C). Then, we conducted the statistical analyses by treating mosquitoes as two experimental groups: mosquitoes fed on *P. relictum*-infected birds (exposed mosquitoes) and mosquitoes fed on uninfected birds (unexposed mosquitoes). Comparisons of the bacterial alpha and beta diversity between groups were conducted in R using packages *phyloseq*, *vegan* (ver 2.6-8)^53^, and *microbiome* (ver. 1.26.0)^54^. Bacterial alpha diversity (within-sample diversity) of ASVs was estimated using observed richness and the Shannon index on samples rarefied to the minimum number of reads present in the samples. The Shannon index accounts for both ASV richness and evenness within a sample, increasing when the number of taxa is higher and more evenly distributed^55^. The Shapiro test^56^ was used to confirm the lack of normality of both observed richness and Shannon index. Kruskal-Wallis tests^57^ were used to statistically test differences in both observed richness and Shannon index by experimental groups (exposed and unexposed mosquitoes). Beta diversity (between-sample diversity) was analysed by a Principal Coordinate Analysis (PCoA) of Bray-Curtis dissimilarity matrix on the ASVs’ relative abundances. PERmutational Multivariate ANalysis Of VAriance (PERMANOVA)^58^ was used to test for differences between the centroids of the experimental groups using adonis2 in *vegan*. Statistical significance of F-values obtained from the PERMANOVA was determined through 999 permutations. The same approaches were used to corroborate the lack of association between bacterial alpha and beta diversity and the variables diet and replicate (Supplementary Material 1). Differences in the relative abundance of different taxa (i.e., microbiota composition) according to experimental groups were assessed at the family and genus levels using the Analysis of Compositions of Microbiomes with Bias Correction 2 (ANCOM-BC2) methodology from the *ancombc* package (ver. 2.6.0)^59^. Briefly, this model consists of a differential abundance analysis of microbiome data through a log-linear regression model while accounting for sample-specific and taxon-specific biases and correcting false positive errors by multi-group comparisons and repeated measurements^59^. Taxa with a prevalence below 10% were filtered out. Holm’s method was used to adjust the p-values for multiple testing. Because mosquitoes in this study fed on different sugar concentrations (0.5%, 2%, and 8%) and originated from three independent replicates, we adjusted the ANCOM-BC2 model for these potential confounders by including them as fixed effects alongside the main variable of interest, parasite exposure. Plots were made using the *ggplot2* package (ver. 3.5.1)^60^.

Additionally, for both datasets with and without *Wolbachia*, we used PICRUSt2 (ver. 2.5.2) to infer the functional profiles of the microbial communities in each sample based on KEGG annotations^61^, obtaining predicted abundances for each KO identifier. In R, we filtered out KOs with a total predicted abundance below 10 across all samples, and compared the predicted abundances between exposed and unexposed mosquitoes using the DESeq2 package (ver. 1.44.0)^62^. According to the *ancombc2* analysis, we adjusted the DESeq2 model for diet and replicate by including them as fixed effects. KOs with a p-value < 0.05 and a |LFC| > 2 were considered differentially abundant between groups. Finally, we retrieved the metabolic pathways associated with each significant KO using the KEGGREST package (ver. 1.44.1)^63^.

## Supplementary Information

Below is the link to the electronic supplementary material.

Supplementary Material 1.

Supplementary Material 2.

Supplementary Material 3.

Supplementary Material 4.

## Data Availability

The datasets generated and analyzed during the current study are available in Digital CSIC: 10.20350/DIGITALCSIC/17367.

## References

[1] WHO. World malaria report 2023: Tracking progress and gaps in the global response to malaria. In World Health Organization. (2023). https://www.who.int/teams/global-malaria-programme/reports/world-malaria-report-2023.

[2] van Riper, C., van Riper, I. I. I., Goff, S. G., Laird, M. & M. L. & The epizootiology and ecological significance of malaria in Hawaiian land birds. *Ecol. Monogr.* **56**(4), 327–344. 10.2307/1942550 (1986).

[3] Lefèvre, T., Vantaux, A., Dabiré, K. R., Mouline, K. & Cohuet, A. Non-genetic determinants of mosquito competence for malaria parasites. *PLoS Pathog* **9**(6), e1003365. 10.1371/journal.ppat.1003365 (2013). 23818841 10.1371/journal.ppat.1003365 PMC3688545

[4] Strand, M. R. Composition and functional roles of the gut microbiota in mosquitoes. *Curr. Opin. Insect Sci.* **28**, 59–65. 10.1016/j.cois.2018.05.008 (2018). 30551768 10.1016/j.cois.2018.05.008 PMC6296257

[5] Coon, K. L., Vogel, K. J., Brown, M. R. & Strand, M. R. Mosquitoes rely on their gut microbiota for development. *Mol. Ecol.* **23**(11), 2727–2739. 10.1111/mec.12771 (2014). 24766707 10.1111/mec.12771 PMC4083365

[6] Wang, Y., Gilbreath, T. M., Kukutla, P., Yan, G., Xu, J. & rd, & Dynamic gut microbiome across life history of the malaria mosquito *Anopheles Gambiae* in Kenya. *PloS One* **6**(9), e24767. 10.1371/journal.pone.0024767 (2011). 21957459 10.1371/journal.pone.0024767 PMC3177825

[7] Muturi, E. J., Dunlap, C., Ramirez, J. L., Rooney, A. P. & Kim, C. H. Host blood-meal source has a strong impact on gut microbiota of *Aedes aegypti*. *FEMS Microbiol. Ecol.* **95**(1). 10.1093/femsec/fiy213 (2019). 10.1093/femsec/fiy213 30357406

[8] Bahia, A. C. et al. Exploring *Anopheles* gut bacteria for *Plasmodium* blocking activity. *Environ. Microbiol.* **16**(9), 2980–2994. 10.1111/1462-2920.12381 (2014). 24428613 10.1111/1462-2920.12381 PMC4099322

[9] Romoli, O. & Gendrin, M. The tripartite interactions between the mosquito, its microbiota and plasmodium. *Parasit. Vectors*. **11**(1). 10.1186/s13071-018-2784-x (2018). 10.1186/s13071-018-2784-x PMC5861617 29558973

[10] Dong, Y., Manfredini, F. & Dimopoulos, G. Implication of the mosquito midgut microbiota in the defense against malaria parasites. *PLoS Pathog.* **5**(5), e1000423. 10.1371/journal.ppat.1000423 (2009). 19424427 10.1371/journal.ppat.1000423 PMC2673032

[11] Martínez-de la Puente, J. et al. Effects of mosquito microbiota on the survival cost and development success of avian *Plasmodium*. *Front. Microbiol.* **11**10.3389/fmicb.2020.562220 (2021). 10.3389/fmicb.2020.562220 PMC7838439 33519724

[12] Cirimotich, C. M. et al. Natural microbe-mediated refractoriness to *Plasmodium* infection in *Anopheles Gambiae*. *Science* **332**(6031), 855–858. 10.1126/science.1201618 (2011). 21566196 10.1126/science.1201618 PMC4154605

[13] Ramirez, J. L. et al. *Chromobacterium* Csp_P reduces malaria and dengue infection in vector mosquitoes and has entomopathogenic and in vitro anti-pathogen activities. *PLoS Pathog*. **10**(10), e1004398. 10.1371/journal.ppat.1004398 (2014). 25340821 10.1371/journal.ppat.1004398 PMC4207801

[14] Zélé, F. et al. *Wolbachia* increases susceptibility to *Plasmodium* infection in a natural system. *Proc. Biol. Sci.* **281**(1779), 20132837. 10.1098/rspb.2013.2837 (2014). 10.1098/rspb.2013.2837 PMC3924077 24500167

[15] Aželytė, J. et al. Anti-microbiota vaccine reduces avian malaria infection within mosquito vectors. *Front. Immunol.* **13**, 841835. 10.3389/fimmu.2022.841835 (2022). 35309317 10.3389/fimmu.2022.841835 PMC8928750

[16] Saab, S. A. et al. The environment and species affect gut bacteria composition in laboratory co-cultured *Anopheles Gambiae* and *Aedes albopictus* mosquitoes. *Sci. Rep.* **10**(1), 3352. 10.1038/s41598-020-60075-6 (2020). 32099004 10.1038/s41598-020-60075-6 PMC7042291

[17] Assentato, L. et al. The type of environment has a greater impact on the larval microbiota of *Anopheles arabiensis* than on the microbiota of their breeding water. *FEMS Microbiol. Ecol.* **101**(1). 10.1093/femsec/fiae161 (2025). 10.1093/femsec/fiae161 PMC11737318 39694819

[18] Martínez-de la Puente, J., Santiago-Alarcon, D., Palinauskas, V. & Bensch, S. *Plasmodium relictum*. *Trends Parasitol.* **37**(4), 355–356. 10.1016/j.pt.2020.06.004 (2021). 32660871 10.1016/j.pt.2020.06.004

[19] Santiago-Alarcon, D., Palinauskas, V. & Schaefer, H. M. Diptera vectors of avian haemosporidian parasites: Untangling parasite life cycles and their taxonomy. *Biol. Rev. Camb. Philos. Soc.* **87**(4), 928–964. 10.1111/j.1469-185X.2012.00234.x (2012). 22616880 10.1111/j.1469-185X.2012.00234.x

[20] Minard, G., Mavingui, P. & Moro, C. V. Diversity and function of bacterial microbiota in the mosquito holobiont. *Parasit. Vectors*. **6**(1), 146. 10.1186/1756-3305-6-146 (2013). 23688194 10.1186/1756-3305-6-146 PMC3667145

[21] Minard, G. et al. Pyrosequencing 16S rRNA genes of bacteria associated with wild tiger mosquito *Aedes albopictus*: A pilot study. *Front. Cell. Infect. Microbiol.* **4**, 59. 10.3389/fcimb.2014.00059 (2014). 24860790 10.3389/fcimb.2014.00059 PMC4030203

[22] Muturi, E. J., Kim, C. H., Bara, J., Bach, E. M. & Siddappaji, M. H. *Culex pipiens* and *Culex restuans* mosquitoes harbor distinct microbiota dominated by few bacterial taxa. *Parasit. Vectors*. **9**(1), 18. 10.1186/s13071-016-1299-6 (2016). 26762514 10.1186/s13071-016-1299-6 PMC4712599

[23] Juma, E. O., Kim, C. H., Dunlap, C., Allan, B. F. & Stone, C. M. *Culex pipiens* and *Culex restuans* egg rafts harbor diverse bacterial communities compared to their midgut tissues. *Parasit. Vectors*. **13**(1), 532. 10.1186/s13071-020-04408-4 (2020). 33109276 10.1186/s13071-020-04408-4 PMC7590256

[24] Dobson, S. L. et al. *Wolbachia* infections are distributed throughout insect somatic and germ line tissues. *Insect Biochem. Mol. Biol.* **29**(2), 153–160. 10.1016/s0965-1748(98)00119-2 (1999). 10196738 10.1016/s0965-1748(98)00119-2

[25] Mejia, A. J., Dutra, H. L. C., Jones, M. J., Perera, R. & McGraw, E. A. Cross-tissue and generation predictability of relative *Wolbachia* densities in the mosquito *Aedes aegypti*. *Parasit. Vectors* **15**(1), 128. 10.1186/s13071-022-05231-9 (2022). 35413938 10.1186/s13071-022-05231-9 PMC9004076

[26] Pidiyar, V. J., Jangid, K., Patole, M. S. & Shouche, Y. S. Studies on cultured and uncultured microbiota of wild *Culex quinquefasciatus* mosquito midgut based on 16s ribosomal RNA gene analysis. *Am. J. Trop. Med. Hyg.* **70**(6), 597–603. 10.4269/ajtmh.2004.70.597 (2004). 15210998

[27] Feng, Y. et al. The microbiota of three *Anopheles* species in China. *J. Am. Mosq. Control Assoc.* **37**(1), 38–40. 10.2987/20-6940 (2021). 33857314 10.2987/20-6940

[28] Arévalo-Cortés, A. et al. Association of midgut bacteria and their metabolic pathways with Zika infection and insecticide resistance in Colombian *Aedes aegypti* populations. *Viruses* **14**(10), 2197. 10.3390/v14102197 (2022). 36298752 10.3390/v14102197 PMC9609292

[29] Garrigós, M. et al. Microbiota composition of *Culex perexiguus* mosquitoes during the West nile virus outbreak in Southern Spain. *PloS One*. **19**(11), e0314001. 10.1371/journal.pone.0314001 (2024). 39556610 10.1371/journal.pone.0314001 PMC11573153

[30] Mandal, R. K. et al. Gut *Bacteroides* act in a microbial consortium to cause susceptibility to severe malaria. *Nat. Commun.* **14**(1). 10.1038/s41467-023-42235-0 (2023). 10.1038/s41467-023-42235-0 PMC10575898 37833304

[31] Yasika, Y. & Shivakumar, M. S. A comprehensive account of functional role of insect gut microbiome in insect orders. *J. Nat. Pest Res.* **11**, 100110. 10.1016/j.napere.2024.100110 (2025).

[32] Oliveira, T. M. P. et al. Bacterial diversity on larval and female *Mansonia* spp. From different localities of Porto Velho, Rondonia, Brazil. *PloS One*. **18**(11), e0293946. 10.1371/journal.pone.0293946 (2023). 38011160 10.1371/journal.pone.0293946 PMC10681206

[33] Capra, E. J. & Laub, M. T. Evolution of two-component signal transduction systems. *Annu. Rev. Microbiol.* **66**(1), 325–347. 10.1146/annurev-micro-092611-150039 (2012). 22746333 10.1146/annurev-micro-092611-150039 PMC4097194

[34] Gutiérrez-López, R. et al. Do mosquitoes transmit the avian malaria-like parasite *Haemoproteus*? An experimental test of vector competence using mosquito saliva. *Parasit. Vectors* **9**(1), 609. 10.1186/s13071-016-1903-9 (2016). 27894354 10.1186/s13071-016-1903-9 PMC5127101

[35] Pigeault, R. et al. Different distribution of malaria parasite in left and right extremities of vertebrate hosts translates into differences in parasite transmission. *Sci. Rep.* **10**(1), 10183. 10.1038/s41598-020-67180-6 (2020). 32576924 10.1038/s41598-020-67180-6 PMC7311528

[36] Palinauskas, V., Valkiūnas, G., Krizanauskiene, A., Bensch, S. & Bolshakov, C. V. *Plasmodium relictum* (lineage P-SGS1): Further observation of effects on experimentally infected passeriform birds, with remarks on treatment with Malarone. *Exp. Parasitol.* **123**(2), 134–139. 10.1016/j.exppara.2009.06.012 (2009). 19545566 10.1016/j.exppara.2009.06.012

[37] Townsend, A. K., Wheeler, S. S., Freund, D., Sehgal, R. N. M. & Boyce, W. M. Links between blood parasites, blood chemistry, and the survival of nestling American crows. *Ecol. Evol.* **8**(17), 8779–8790. 10.1002/ece3.4287 (2018). 30271545 10.1002/ece3.4287 PMC6157653

[38] Pakpour, N., Akman-Anderson, L., Vodovotz, Y. & Luckhart, S. The effects of ingested mammalian blood factors on vector arthropod immunity and physiology. *Microbes Infect.* **15**(3), 243–254. 10.1016/j.micinf.2013.01.003 (2013). 23370408 10.1016/j.micinf.2013.01.003 PMC3602389

[39] Gutiérrez-López, R., Martínez-de la Puente, J., Gangoso, L., Soriguer, R. C. & Figuerola, J. Comparison of manual and semi-automatic DNA extraction protocols for the barcoding characterization of hematophagous louse flies (Diptera: *Hippoboscidae*). *J. Vector Ecol.: J. Soc. Vector Ecol.* **40**(1), 11–15. 10.1111/jvec.12127 (2015). 10.1111/jvec.12127 26047179

[40] Hellgren, O., Waldenström, J. & Bensch, S. A new PCR assay for simultaneous studies of *Leucocytozoon*, *Plasmodium*, and *Haemoproteus* from avian blood. *J. Parasitol.* **90**(4), 797–802. 10.1645/GE-184R1 (2004). 15357072 10.1645/GE-184R1

[41] Kearse, M. Geneious basic: An integrated and extendable desktop software platform for the organization and analysis of sequence data. *Bioinformatics* **28**(12), 1647–1649. 10.1093/bioinformatics/bts199 (2012). 22543367 10.1093/bioinformatics/bts199 PMC3371832

[42] Bensch, S., Hellgren, O. & Pérez-tris, J. MalAvi: A public database of malaria parasites and related haemosporidians in avian hosts based on mitochondrial cytochrome b lineages. *Mol. Ecol. Resour.* **9**(5), 1353–1358. 10.1111/j.1755-0998.2009.02692.x (2009). 21564906 10.1111/j.1755-0998.2009.02692.x

[43] García-Ruiz, O. et al. Sugar diet affects *Culex pipiens* early-life mortality, biochemical parameters, and immunocompetence. *Ecosphere* **16**(4). 10.1002/ecs2.70158 (2025).

[44] Schaffner, E. et al. The mosquitoes of Europe: An identification and training programme. Paris (FRA); Montpellier: IRD; EID, 1 CD ROM. (Didactiques). (2001).

[45] Kazlauskienė, R., Bernotienė, R., Palinauskas, V., Iezhova, T. A. & Valkiūnas, G. *Plasmodium relictum* (lineages pSGS1 and pGRW11): Complete synchronous sporogony in mosquitoes *Culex pipiens pipiens*. *Exp. Parasitol.* **133**(4), 454–461. 10.1016/j.exppara.2013.01.008 (2013). 23337824 10.1016/j.exppara.2013.01.008

[46] Binetruy, F., Dupraz, M., Buysse, M. & Duron, O. Surface sterilization methods impact measures of internal microbial diversity in ticks. *Parasit. Vectors* **12**(1), 268. 10.1186/s13071-019-3517-5 (2019). 31138324 10.1186/s13071-019-3517-5 PMC6537145

[47] Andrews, S. FastQC: A quality control tool for high throughput sequence data (2020).

[48] Ewels, P., Magnusson, M., Lundin, S. & Käller, M. MultiQC: Summarize analysis results for multiple tools and samples in a single report. *Bioinformatics* **32**(19), 3047–3048. 10.1093/bioinformatics/btw354 (2016). 27312411 10.1093/bioinformatics/btw354 PMC5039924

[49] Core Team, R. R: A Language and environment for statistical computing. *R Foundation Stat. Comput.* (Vienna Austria, 2022). https://www.R-project.org/.

[50] Callahan, B. J. et al. DADA2: High-resolution sample inference from illumina amplicon data. *Nat. Methods* **13**(7), 581–583. 10.1038/nmeth.3869 (2016). 27214047 10.1038/nmeth.3869 PMC4927377

[51] Quast, C. et al. The SILVA ribosomal RNA gene database project: improved data processing and web-based tools. *Nucleic Acids Res.* **41**, D590–D596. 10.1093/nar/gks1219 (2013). 23193283 10.1093/nar/gks1219 PMC3531112

[52] McMurdie, P. J. & Holmes, S. Phyloseq: An R package for reproducible interactive analysis and graphics of Microbiome census data. *PloS One*. **8**(4), e61217. 10.1371/journal.pone.0061217 (2013). 23630581 10.1371/journal.pone.0061217 PMC3632530

[53] Oksanen, J. et al. vegan: Community Ecology Package_ R package version 2.6-8. (2024). https://CRAN.R-project.org/package=vegan.

[54] Lahti, L. & Shetty, S. microbiome R package. (2012–2019). http://microbiome.github.io.

[55] Shannon, C. E. A mathematical theory of communication. *BSTJ* **27**(3), 379–423. 10.1002/j.1538-7305.1948.tb01338.x (1948).

[56] Shapiro, S. S. & Wilk, M. B. An analysis of variance test for normality (complete samples). *Biometrika* **52**(3–4), 591–611. 10.1093/biomet/52.3-4.591 (1965).

[57] Kruskal, W. H. & Wallis, W. A. Use of ranks in one-criterion variance analysis. *J. Am Stat. Assoc.* **47**(260), 583. 10.2307/2280779 (1952).

[58] Anderson, M. J. A new method for non-parametric multivariate analysis of variance: Non-parametric manova for ecology. *Austral Ecol.* **26**(1), 32–46. 10.1111/j.1442-9993.2001.01070.pp.x (2001).

[59] Lin, H. & Peddada, S. D. Analysis of compositions of microbiomes with bias correction. *Nat. Commun.* **11**(1), 3514. 10.1038/s41467-020-17041-7 (2020). 32665548 10.1038/s41467-020-17041-7 PMC7360769

[60] Wickham, H. *ggplot2: Elegant Graphics for Data Analysis* (Springer, 2016).

[61] Langille, M. G. I. et al. Predictive functional profiling of microbial communities using 16S rRNA marker gene sequences. *Nat. Biotechnol.* **31**(9), 814–821. 10.1038/nbt.2676 (2013). 23975157 10.1038/nbt.2676 PMC3819121

[62] Love, M. I., Huber, W. & Anders, S. Moderated estimation of fold change and dispersion for RNA-seq data with DESeq2. *Genom. Biol.* **15**(12), 550. 10.1186/s13059-014-0550-8 (2014). 10.1186/s13059-014-0550-8 PMC4302049 25516281

[63] Tenenbaum, D. KEGGREST Client-side REST access to the Kyoto encyclopedia of genes and genomes (KEGG). 10.18129/B9.bioc.KEGGREST (2024).

