# Supplementary material for: Effects of avian Plasmodium exposure on the microbiota of Culex pipiens
Source: Sci Rep. 2025 Nov 6;15:38898. doi: 10.1038/s41598-025-22774-w (PMC12592391; doi:10.1038/s41598-025-22774-w)
Supplement: Supplementary file 4 — Supplementary Material 4. [file 41598_2025_22774_MOESM4_ESM.docx]

**Effects of avian *Plasmodium* exposure on the microbiota of *Culex pipiens***

Marta Garrigós^1,2*^, Olaya García-Ruiz^1,2^, Charlotte R. Enkvist^3^, María José García-López^4,5,6^, Isabel Moreno-Indias^4,7^, María José Ruiz-López^1,2^, Jesús Veiga^1^, Jordi Figuerola^1,2^, Elin Videvall^3^, Josué Martínez-de la Puente^1,2^

1. Department of Conservation Biology and Global Change, Doñana Biological Station (EBD), CSIC, Sevilla, Spain.
2. CIBER de Epidemiología y Salud Pública (CIBERESP), Madrid, Spain.
3. Department of Ecology and Genetics, Uppsala University, Uppsala, Sweden.
4. Department of Endocrinology and Nutrition, Instituto de Investigación Biomédica de Málaga (IBIMA).
5. Hospital Universitario Virgen de la Victoria, Málaga, Spain.
6. Facultad de Medicina, Universidad de Málaga, Málaga, Spain.
7. CIBER de Fisiopatología de la Obesidad y Nutrición (CIBEROBN), Madrid, Spain.

* E-mail: [marta.garrigos@ebd.csic.es](mailto:marta.garrigos@ebd.csic.es)

1. Lack of association between bacterial alpha- and beta-diversity and the variables diet and replicate.

To check for the potential association between alpha- and beta-diversities and the variables diet and replicate, we used Kruskal-Wallis tests and PERmutational Multivariate ANalysis Of VAriance (PERMANOVA), respectively. Statistical significance of F-values obtained from the PERMANOVA was determined by comparison to 999 permutations.

We corroborated the lack of significant associations in both cases. No significant differences were found in observed Richness by diet (Kruskal-Wallis: χ² = 1.053, Degrees of freedom (Df)= 2, P = 0.591) nor replicate (K-W: χ² = 1.217, Df = 2; P = 0.544). Similarly, Shannon indexes estimates were not significantly related to diet (Kruskal-Wallis: χ² = 1.191, Df = 2, P = 0.551) nor replicate (K-W: χ² =, Df = 2; P = 0.438). PERMANOVA also showed lack of significant association between beta-diversity and both diet (PERMANOVA: F= 2.884, Df = 2, P = 0.076) and replicate (PERMANOVA: F= 1.589, Df =

2, P = 0.181).

1. Exploratory comparison of *Cx. pipiens* abdominal microbiota by infection status.

Overall, infected mosquitoes apparently showed a higher abundance of bacteria of the phylum Firmicutes than uninfected and unexposed ones (Figure S1). At the genus level, infected mosquitoes showed a higher relative abundance of bacteria of the genera *Faecalibacterium* and *Vagococcus* and a lower relative abundance of *Stenotrophomonas* than uninfected and unexposed mosquitoes (Figure S5). Similarly, infected mosquitoes presented a higher bacterial alpha-diversity, including observed richness and Shannon index estimated, than the other groups (Figure S8A). However, these differences may be due to one of the two infected mosquitoes included in the analysis, with the other one being more similar to the uninfected and unexposed samples (Figures S1-S5). This is supported by the PCoA plot, in which the two infected samples did not cluster together (Figure S8B).

1. Supplementary Figures

**
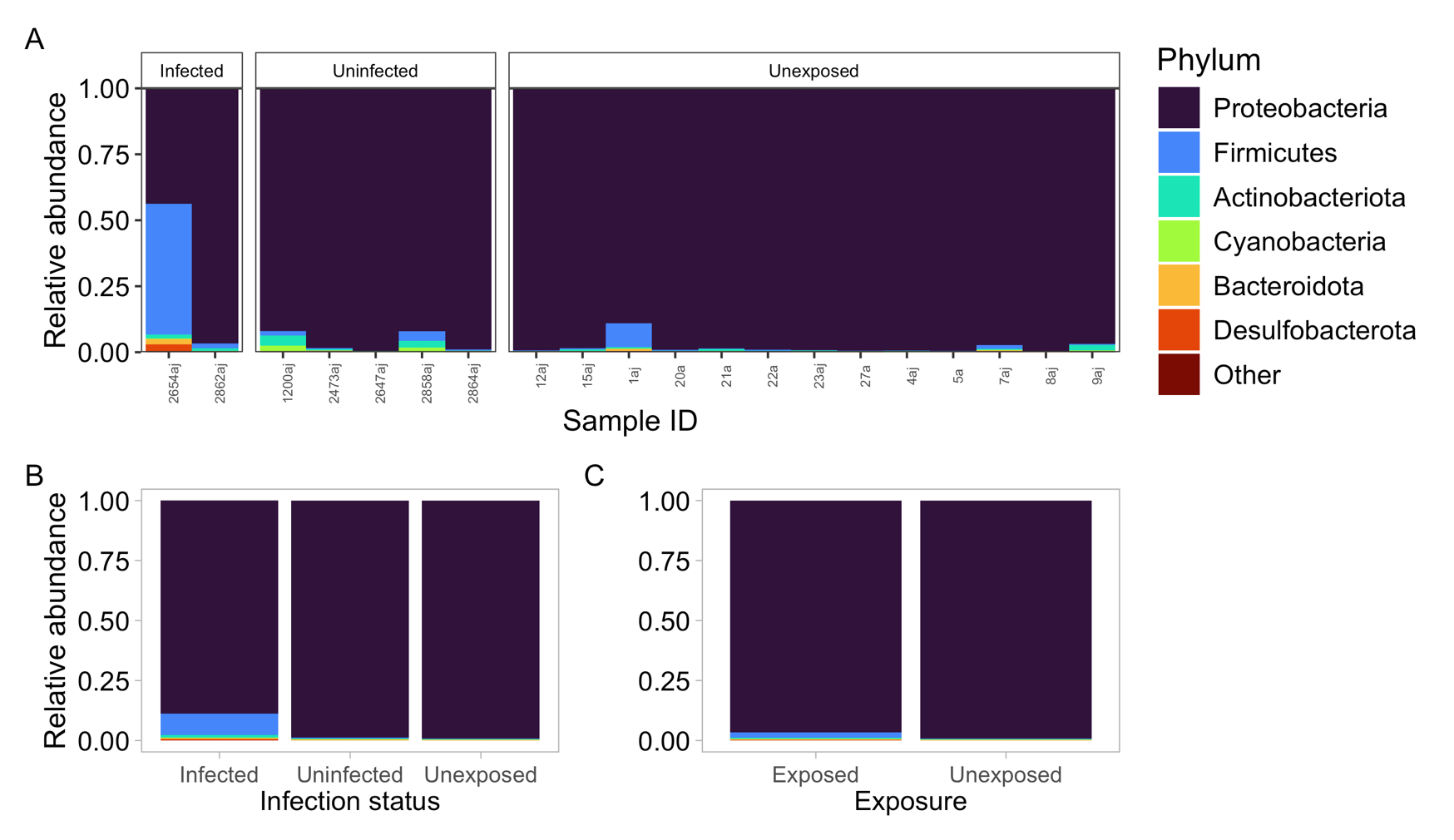
**

**Figure S1.** Relative abundance of most abundant phyla for: A. Individual samples separated by infection status (Infected: mosquitoes that fed on a *P. relictum*-infected bird and became infected; Uninfected: mosquitoes that fed on a *P. relictum*-infected bird and did not become infected; Unexposed: mosquitoes that fed on an uninfected bird); B. Samples grouped by Infection status; C. Samples grouped by exposure.


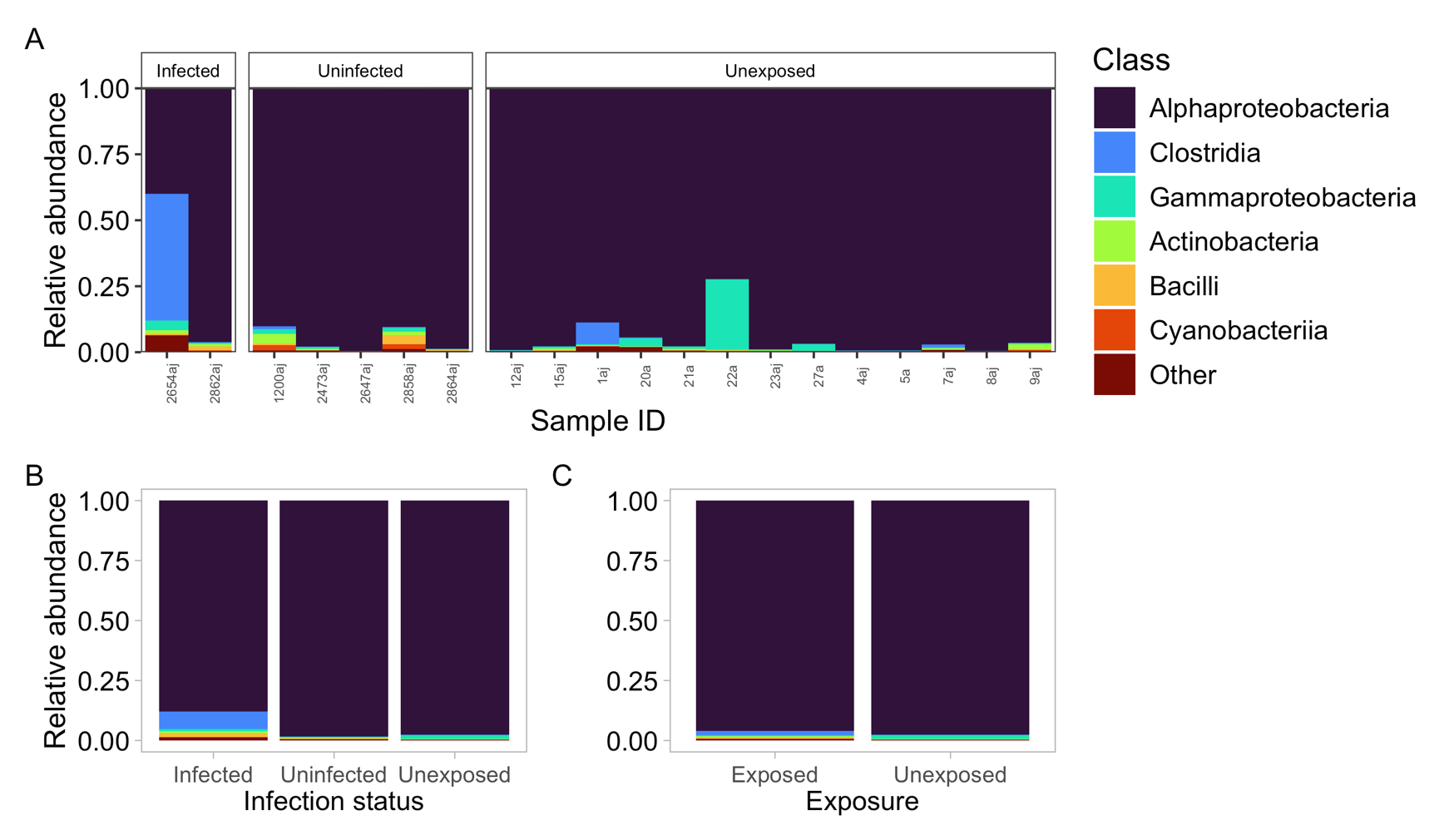


**Figure S2.** Relative abundance of most abundant classes for: A. Individual samples separated by infection status (Infected: mosquitoes that fed on a *P. relictum*-infected bird and became infected; Uninfected: mosquitoes that fed on a *P. relictum*-infected bird and did not become infected; Unexposed: mosquitoes that fed on an uninfected bird); B. Samples grouped by Infection status; C. Samples grouped by exposure.

**
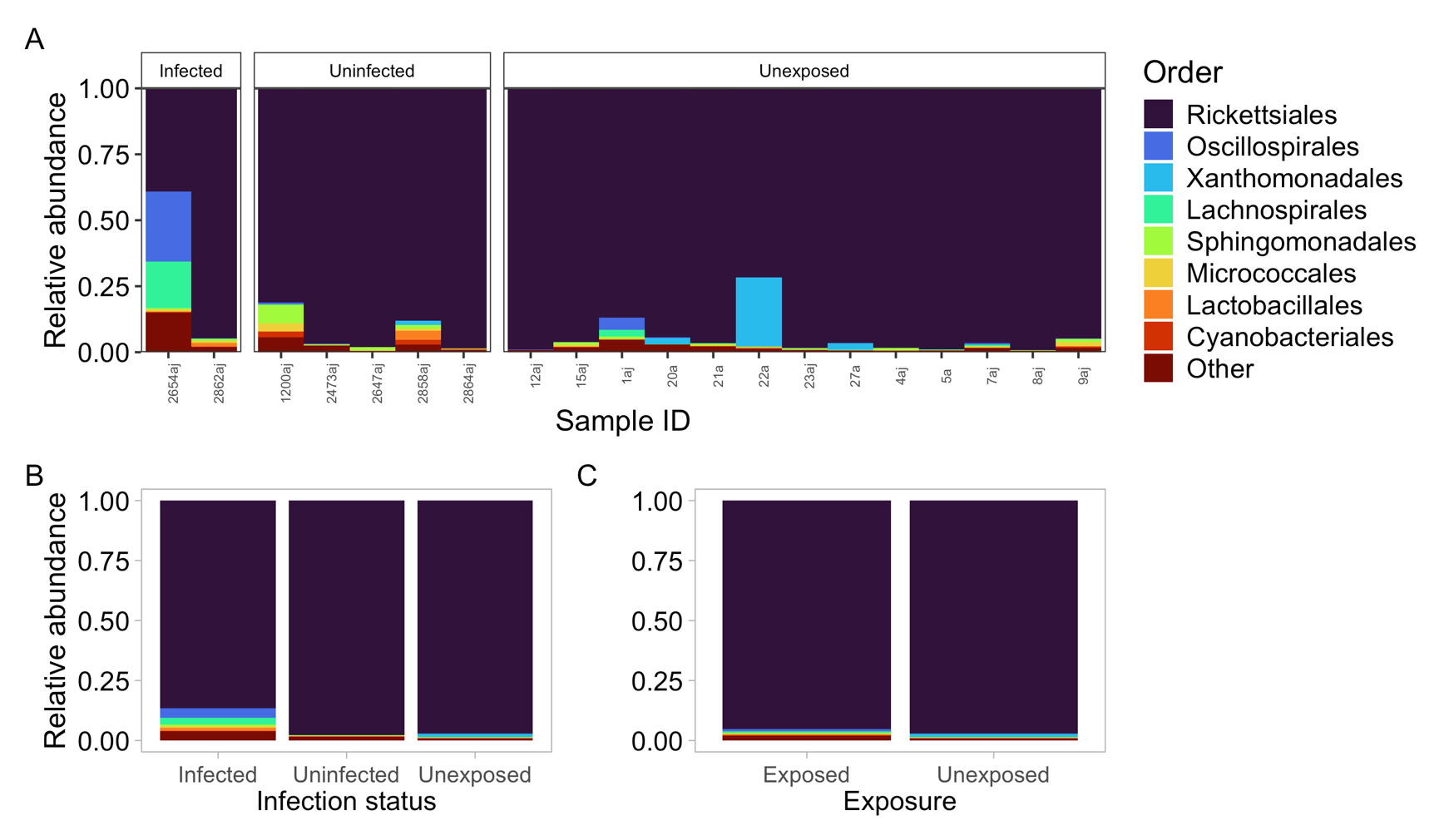
Figure S3.** Relative abundance of most abundant orders for: A. Individual samples separated by infection status (Infected: mosquitoes that fed on a *P. relictum*-infected bird and became infected; Uninfected: mosquitoes that fed on a *P. relictum*-infected bird and did not become infected; Unexposed: mosquitoes that fed on an uninfected bird); B. Samples grouped by Infection status; C. Samples grouped by exposure.

**
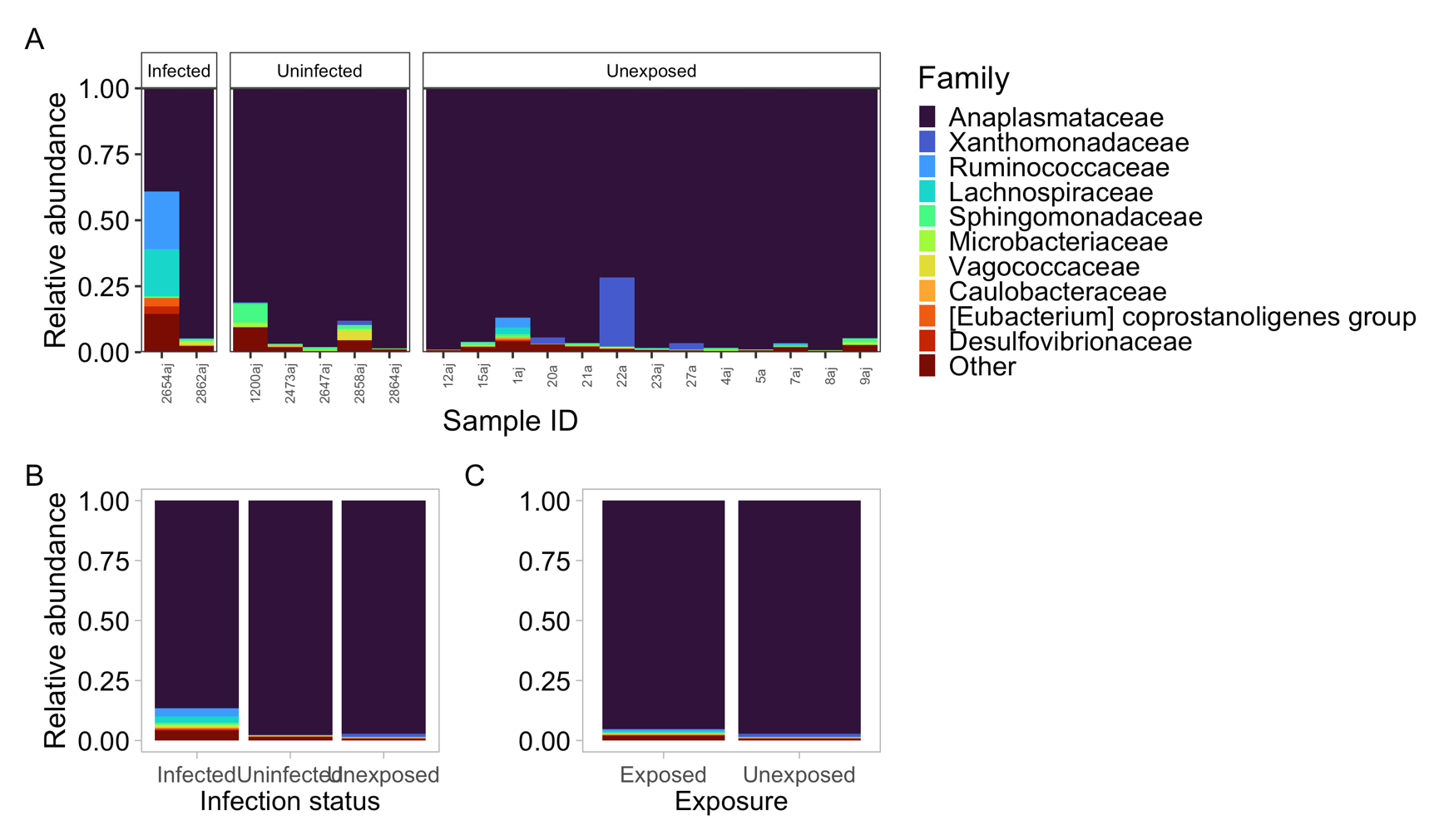
Figure S4.** Relative abundance of most abundant families for: A. Individual samples separated by infection status (Infected: mosquitoes that fed on a *P. relictum*-infected bird and became infected; Uninfected: mosquitoes that fed on a *P. relictum*-infected bird and did not become infected; Unexposed: mosquitoes that fed on an uninfected bird); B. Samples grouped by Infection status; C. Samples grouped by exposure.


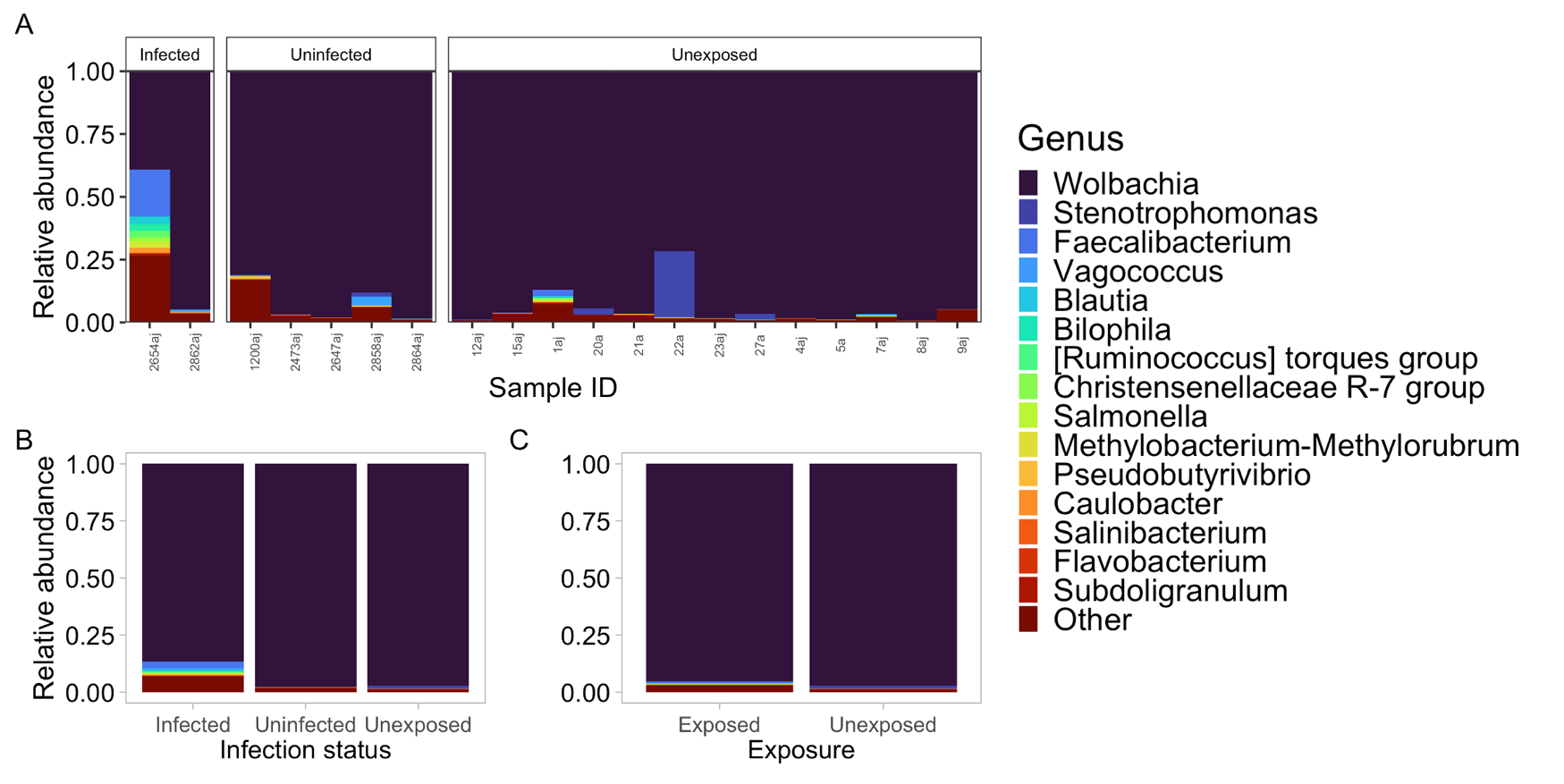


**Figure S5.** Relative abundance of most abundant genera for: A. Individual samples separated by infection status (Infected: mosquitoes that fed on a *P. relictum*-infected bird and became infected; Uninfected: mosquitoes that fed on a *P. relictum*-infected bird and did not become infected; Unexposed: mosquitoes that fed on an uninfected bird); B. Samples grouped by Infection status; C. Samples grouped by exposure.


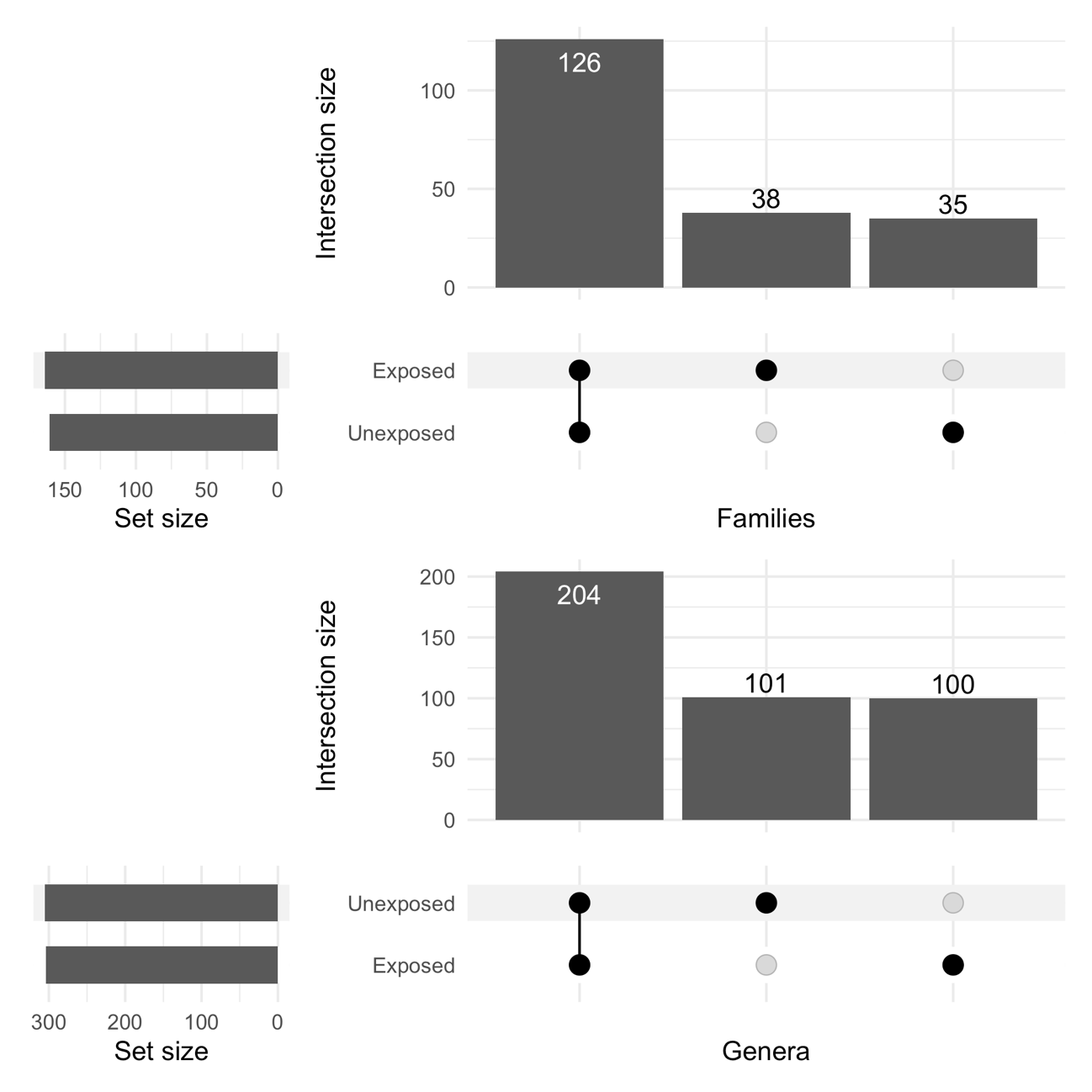


**Figure S6.** Upsetplot with overlapping taxa between mosquitoes exposed to *P. relictum* infected birds (exposed) and to uninfected birds (unexposed) at family (up) and genus (down) levels.


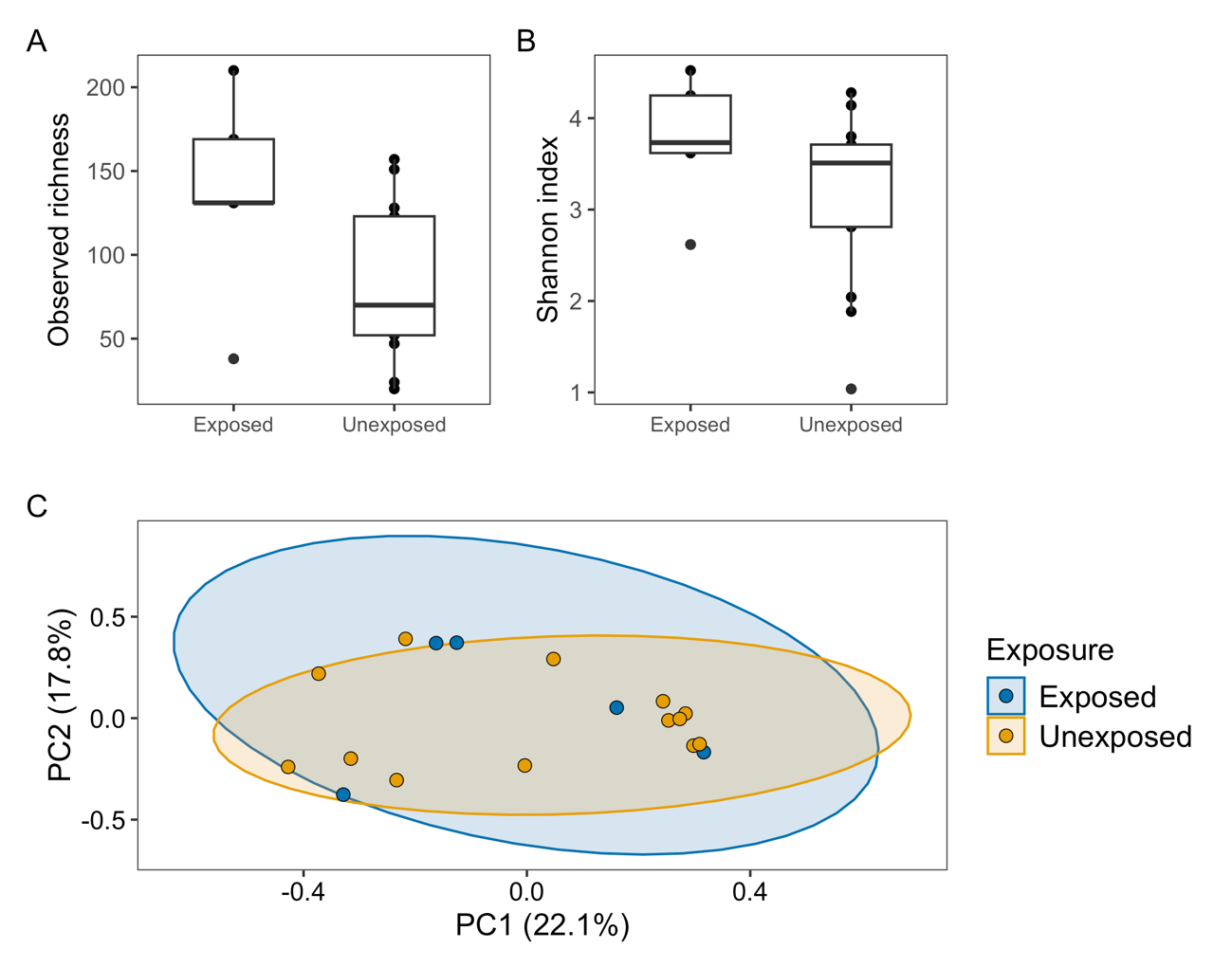


**Figure S7.** Diversity of abdominal microbiota of mosquitoes exposed to *Plasmodium relictum* infected birds (exposed; blue) and to uninfected bird (unexposed; yellow), excluding *Wolbachia* from the analyses. A. Distribution of the observed richness in exposed and unexposed mosquitoes. The horizontal line represents the median observed richness of each group. The boxes delimit the upper and lower quartiles, and the vertical lines go from the lower and upper quartiles to the minimum or maximum values. B. Distribution of the Shannon index estimate in exposed and unexposed mosquitoes. C. Beta-diversity. Principal co-ordinate analysis (PCoA) for Bray-Curtis distance matrix (relative abundance) in exposed and unexposed mosquitoes. Percentages shown in PC1 and PC2 axes refer to the percentage of variation explained by each of the two selected main coordinate axes.


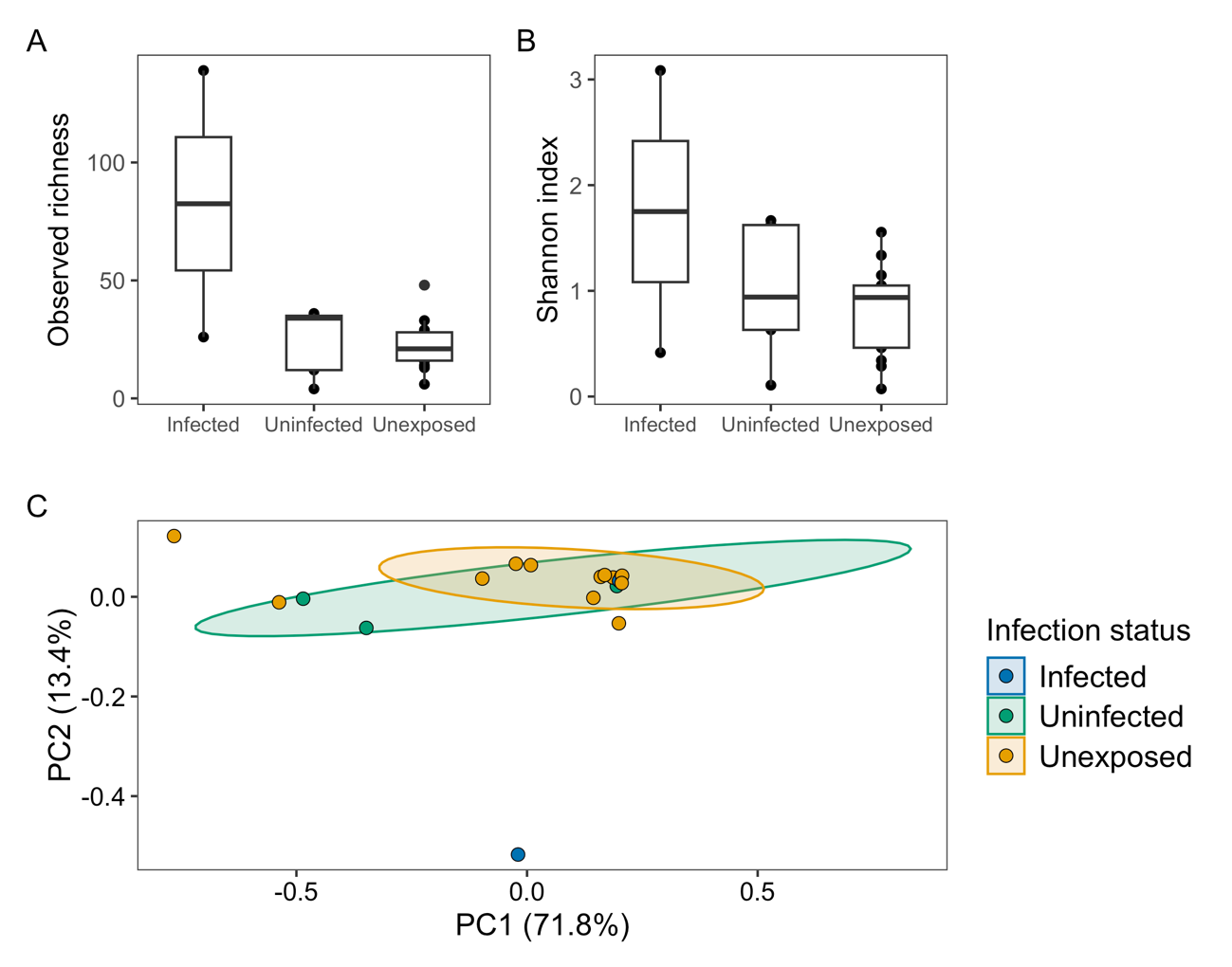


**Figure S8.** Diversity of the abdominal microbiota of infected mosquitoes exposed to *Plasmodium relictum* infected birds (infected; blue), uninfected mosquitoes exposed to *Plasmodium relictum* infected birds (uninfected; yellow), and mosquitoes exposed to uninfected bird (unexposed; green). A. Distribution of the observed richness in infected, uninfected and unexposed mosquitoes. The horizontal line represents the median observed richness of each group. The boxes delimit the upper and lower quartiles, and the vertical lines go from the lower and upper quartiles to the minimum or maximum values. B. Distribution of the Shannon index estimate in infected, uninfected and unexposed mosquitoes. C. Beta-diversity. Principal co-ordinate analysis (PCoA) for Bray-Curtis distance matrix (relative abundance) in infected, uninfected and unexposed mosquitoes. Percentages shown in PC1 and PC2 axes refer to the percentage of variation explained by each of the two selected main coordinate axes. Note that one infected mosquito overlaps with dots of other groups and it is not clearly shown.
